# The design of a multifunctional separator regulating the lithium ion flux for advanced lithium-ion batteries†

**DOI:** 10.1039/c9ra08006f

**Published:** 2019-12-04

**Authors:** Guohua Sun, Jiacong Guo, Hongqing Niu, Nanjun Chen, Mengying Zhang, Guofeng Tian, Shengli Qi, Dezhen Wu

**Affiliations:** State Key Laboratory of Chemical Resource Engineering, Beijing University of Chemical Technology Beijing 100029 China wdz@mail.buct.edu.cn +86 10 6442 1693; Changzhou Institute of Advanced Materials, Beijing University of Chemical Technology Changzhou 213164 Jiangsu China qisl@mail.buct.edu.cn +86 10 6442 2381; Department of Energy Engineering, College of Engineering, Hanyang University Seoul 04763 Republic of Korea

## Abstract

Herein, we design a controllable approach for preparing multifunctional polybenzimidazole porous membranes with superior fire-resistance, excellent thermo-stability, and high wettability. Specifically, the recyclable imidazole is firstly utilized as the eco-friendly template for micropores formation, which is an interesting finding and has tremendous potential for low-cost industrial production. The unique backbone structure of the as-prepared polybenzimidazole porous membrane endows the separator with superb thermal dimensional stability at 300 °C. Most significantly, the inherent flame retardancy of polybenzimidazole can ensure the high security of lithium-ion batteries, and the existence of polar groups of imidazole can regulate the Li^+^ flux and improve the ionic conductivity of lithium ions. Notably, the cell with a polybenzimidazole porous membrane presents higher capability (131.7 mA h g^−1^) than that of a commercial Celgard membrane (95.4 mA h g^−1^) at higher charge–discharge density (5C), and it can work normally at 120 °C. The fascinating comprehensive properties of the polybenzimidazole porous membrane with excellent thermal-stability, satisfying wettability, superb flame retardancy and good electrochemical performance indicate its promising application for high-safety and high-performance lithium-ion batteries.

## Abstract 2

A multifunctional PBI porous membrane with superior fire-resistance, excellent thermo-stability and high wettability is designed.

## Graphical abstract

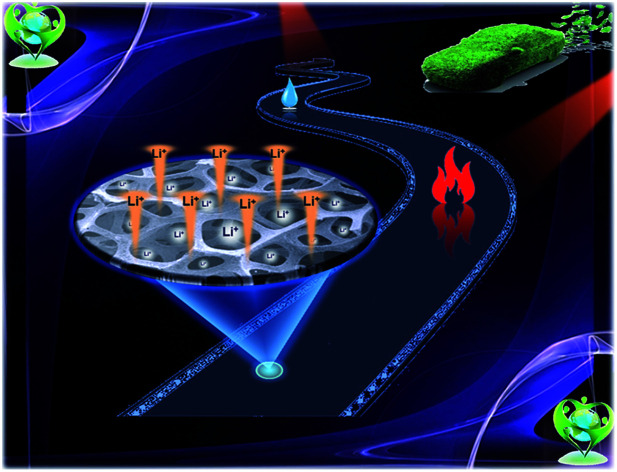

## Introduction

The rapidly increasing demand for high-energy density greatly promotes the development of electrochemical energy storage devices.^1–5^ Lithium-ion batteries (LIBs) have affected the development of the portable electronics market, and also dominate the large applications of electric vehicles and grid scale energy storage systems.^6–9^ The safety issue of batteries, however, has attracted increasing attention with a series of accidents occurring, which can be caused by the poor thermal dimensional stability of the separator.^10,11^ The rational design of cell components is very important to obtain satisfying LIBs. In LIBs, the separator possess crucial functions of physically separating the anode and cathode and enabling free transport of lithium ions,^12,13^ affecting the electrochemical performance of batteries.^14,15^ Notably, the internal short-circuits could be produced under abnormal conditions such as overcharging and high-temperature.^16,17^ Therefore, the excellent thermal stability of a separator is highly important to avoid thermal runaway accompanied by fire and even explosion. The polyolefin-based (PE and PP) membranes are widely used as the commercial separators for LIBs owing to their excellent mechanical strength and acceptable cost.^18,19^ However, the major drawbacks of PO microporous membranes lie in their low porosity and hydrophobic nature. Besides, the poor thermal-stability of PO microporous membranes limits their further development in high-performance batteries because of their low melting points (PE/130 °C, PP/160 °C).^20,21^ To overcome the aforementioned issues, numerous strategies have been intensively investigated to obtain superior separators by introducing inorganic particles into or onto the PO separators such as aluminum oxide,^22–24^ silicon dioxide,^25–27^ and titanium dioxide,^28,29^ significantly improving the thermal-stability and the wettability of the separator. Additionally, the various thermal resistance polymers containing polyetherimide separator,^30^ poly(phthalazinone ether sulfone ketone) separator,^31^ and polyimide separator have also been widely used as heat-resistant skeleton.^32,33^ Although the mentioned separators showed good thermal-stability, the flame retardancy of separator still need to be further improved. Polybenzimidazole (PBI) possessing superior thermal resistance and good mechanical property is one kind of aromatic polymers.^34–36^ Even more remarkable, the PBI presents outstandingly inherent flame retardancy due to the unique backbone structure and satisfying wettability on account of the polar group of the imidazole ring. Meanwhile, the existence of polar groups of N–H bonds can improve the migration rate of lithium ions.^37,38^ These results indicate that PBI has the tremendous application potential in LIBs.

Herein, the multifunctional PBI porous membrane with superior fire-resistance, excellent thermo-stability and high wettability is designed and fabricated *via* a novel and controllable approach of simply extracting the imidazole from dry PBI blend membranes with deionized water. The recyclable imidazole, for the first time to our knowledge, is utilized as the template for micropores formation, which method of pore formation is highly efficient and eco-friendly to fabricate the separator with high porosity and easy for large scale production. Our results demonstrate that the as-prepared PBI porous membrane is a very promising separator owing to the good electrochemical performances, excellent thermal-stability, and satisfying wetting property. Most importantly, the outstanding flame retardancy of PBI porous membranes can internally improve the critical safety issues of LIBs, and the existence of polar groups of PBI can regulate the Li^+^ flux and improve the ionic conductivity of lithium ions.

## Experimental

### Materials

3,3′-diaminobenzidine (DAB), polyphosphoric acid (PPA, >85% P_2_O_5_), and lithium chloride were supplied by J&K Scientific Ltd, China. Isophthalic acid (IPA) was purchased from Aladdin Bio-Chem Technology Co., Ltd, Shanghai, China. Diethyl carbonate (DEC), *N*,*N*′-dimethylacetamide (DMAc), dimethyl carbonate (DMC), and ethylene carbonate (EC) were directly used and supplied by Energy Chemical, Shanghai, China. The Celgard-2400 membrane is used as the separator of LIBs as contrastive research.

### Synthesis of PBI

The PBI was prepared by the microwave irradiation equipment as reported in the literature.^39–41^ The synthetic route of PBI is displayed in Fig. S1,† and the representative synthetic route has been introduced as show below. First, the 50 g polyphosphoric acids (PPA) need to deaerate under N_2_ atmosphere by mechanical stirring at 100 °C for 20 min under N_2_ atmosphere. Subsequently, the 1.4082 g 3,3′-diaminobenzidine (DAB) and the 1.0918 g isophthalic acid (IPA) were put into PPA under the mechanical stirring and N_2_ atmosphere. The polymerization reaction was programmed at 200 W using the following condition: 110 °C for 15 min, 120 °C for 15 min, 140 °C for 90 min, 170 °C for 20 min, 200 °C for 200 min. Then, the viscous polymer solution was poured into the deionized water to precipitate the polymer. Furthermore, the precipitate would be washed using DI water and saturated sodium hydrogen carbonate solution. The precipitate also need be washed using the acetone for removing the impurities. At last, the dry polymer was obtained by filtering and drying at 60 °C in a vacuum for 10 h. ^1^H NMR (400 MHz, DMSO-d_6_) *δ*: 13.31 (s, 2H, N–H), 9.19 (s, 1H, Ar), 8.35 (d, *J* = 10.2 Hz, 2H, Ar), 8.07 (s, 1H, Ar), 7.86 (d, *J* = 10.2 Hz, 2H, Ar), 7.81 (s, 1H, Ar), 7.71–7.66 (m, 3H, Ar) (Fig. S2†).

### Fabrication of PBI porous membranes

The PBI porous membranes were fabricated using the imidazole as the eco-friendly template. First, PBI (0.9 g) and different amount of imidazole (1.0 g, 2.0 g, 3.0 g) were dissolved by heating reflux in 10 mL of DMAC. The homogeneous solution was obtained after filtering insoluble matter. Afterward, the polymer solution was poured into the glass culture dish and dried to remove the solvent by keeping the temperature of 80 °C for 12 h. The as-prepared blend membrane need to remove the imidazole by immersing into deionized water at 50 °C for 10 h, and the imidazole can be used by recycling. Then the sample was dried in an oven at 75 °C for 12 h to acquire the dry membrane. For different amount of imidazole (1.0 g, 2.0 g, 3.0 g), the as-obtained PBI porous membrane are labeled as PBI-1, PBI-2, and PBI-3, respectively.

### Materials characterization

Morphology of PBI porous membranes were examined employing a scanning electron microscopy (SEM) (S-4700, Hitachi). The mechanical strength was obtained by electronic universal testing machine with a strain rate of 10 mm min^−1^. The chemical composition of the sample was analyzed using FT-IR (Nicolet 8700). The thermal dimensional stability was investigated under tensile mode by the thermal mechanical analysis (TMA, TA Q800). Thermogravimetric analysis (TGA) was performed to investigate the thermal-stability from 25 to 800 °C, which was conducted under air atmosphere at 5 °C min^−1^. The wetting property of the sample was evaluated by a contact angle goniometer (JC2000D2M, Powereach). Differential scanning calorimetry was applied to detect the melting point of membrane under N_2_ atmosphere from 30 to 300 °C at a heating rate of 10 °C min^−1^. The sample was immersed in the liquid electrolyte (EC/DMC/DEC, 1 : 1 : 1, v/v) for 2 h to measure the liquid electrolyte uptake. The membranes were weighed immediately after removing free surface solution.^42^ The liquid electrolyte uptake was evaluated using eqn (1):

1 Uptake = (*W*_1_ − *W*_0_)/*W*_0_ × 100%

where the *W*_0_ and *W*_1_ are the mass of the dry separator and soaked separator, respectively.

The porosity of membrane was measured using *n*-butanol uptake method,^43^ and then it was evaluated using eqn (2):

2 Porosity = (*m*_b_/*ρ*_b_)/(*m*_b_/*ρ*_b_ + *m*_p_/*ρ*_p_) × 100%

where *m*_b_ and *m*_p_ are the mass of the *n*-butanol and the dry separator, the *ρ*_b_ and *ρ*_p_ are the density of *n*-butanol and polymer.

### Electrochemical measurements

The coin cell was obtained by sandwiching a separator between the lithium metal foil anode and the LiFePO_4_ cathode in an argon-filled glove box, followed by filling with the electrolyte. The charge–discharge curves and capacities of batteries were evaluated at room temperature by battery tester, and the cycling stability was tested at 25 °C and 120 °C. The electrochemical impedance spectroscopy (EIS) of the separator was recorded to evaluate the ionic conductivity of PBI porous membrane.^44,45^ In addition, the battery with a Celgard separator was also tested for contrastive research. The impedance measurement was conducted with frequency range from 1 Hz to 100 kHz. And, the ionic conductivity could be evaluated based on the eqn (3):

3 *σ* = *d*/(*R*_b_*S*)

where *d* is the thickness of the membrane, *R*_b_ is the bulk resistance of liquid electrolyte-soaked membrane and *S* is the cross-sectional area between membrane.

## Results and discussion

**Fig. 1:**
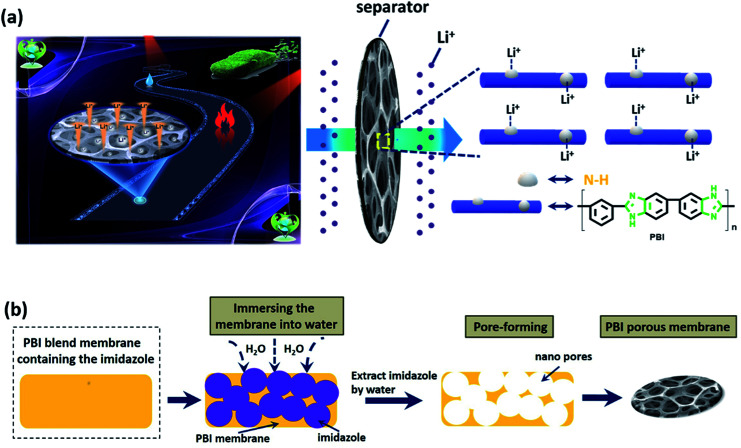
(a) Schematic diagram of the interaction between the polar functional groups and Li ions; (b) the illustrative procedures for the formation of PBI porous membrane.

**Fig. 2:**
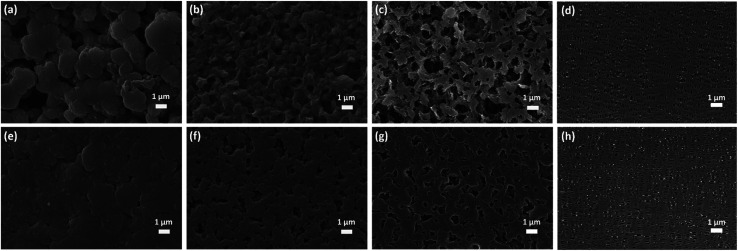
SEM images of the two sides (d and h) of Celgard membrane and the air side surface (a–c) and glass side surface (e–g) of PBI porous membranes: (a and e) PBI-1, (b and f) PBI-2, (c and g) PBI-3.

**Fig. 3:**
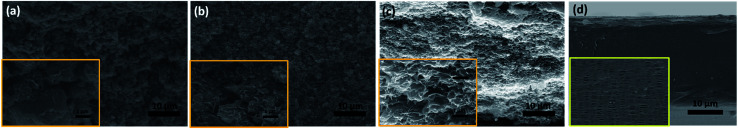
Cross-section morphology and the magnified cross section morphology of (a) PBI-1, (b) PBI-2, (c) PBI-3 membranes, and (d) Celgard membrane.

The FTIR spectrum of the PBI porous membrane is displayed in Fig. S3.† The characteristic absorption band of membrane from 2520 to 3729 cm^−1^ corresponds to the N–H bonds of PBI main chain. The band at 1630 cm^−1^ is be ascribed to the stretching vibration of C=N bond. The characteristic peaks of FTIR spectrum well match with the as-synthesized PBI membrane, which provides strong evidence for the composition of the PBI porous membrane. The sponge-like micropores of PBI membrane is obtained using the imidazole as the eco-friendly template. As schematically indicated in Fig. 1b, the imidazole dispersing inside the membrane is easily removed from the water to form the large quantities of micropores. The ion transport channels is a vital factor for improving the performance of supercapacitor and battery.^46–48^ These micropores can act as the channels to regulate the Li^+^ flux and transport the lithium-ion since the large quantity of polar groups of N–H bonds can provide high-concentration functional sites for the efficient adhesion and homogeneous distribution of Li ions,^37^ as shown in Fig. 1a. Besides, the interaction between the polar ether bonds of electrolyte solvent (*i.e.*, EC, DMC, DEC) and the nitrogen atoms (*e.g.*, in the PBI backbone) can improve the compatibility of the separator with the electrolyte,^49–53^ which can enhance the hydrophilic performance of the membrane and consequently the electrochemical performance of the LIB. Fig. 2 displays the surface SEM images of the as-synthesized PBI porous membranes. It's obvious that different pore morphologies are prepared by adjusting imidazole contents. The PBI-1 membrane shows a small number of micropores. Nonetheless, with the further increase of the content of imidazole, the numerous interconnected pores of the PBI-3 membrane are formed on their air side surfaces. For all the membranes, we can clearly observe the existence of micro holes on their air side surfaces, whereas only PBI-3 membrane reveals satisfying micropores on its glass side surface. Besides, although the surface morphologies of PBI-3 membrane are different on the two sides, the pore sizes are all in the micron range and well-distributed at the surface of membrane. To observe the overall film uniformity and pore-size distribution inside the film, the cross-section and the magnified cross-section images of PBI porous membrane are shown in Fig. 3. The PBI-3 membrane exhibits a sponge-like and interconnected porous structure occupying the whole cross section, which enables the easy transportation of lithium ions. Thus, PBI-3 membrane is preferred as a separator in the following test.

**Fig. 4:**
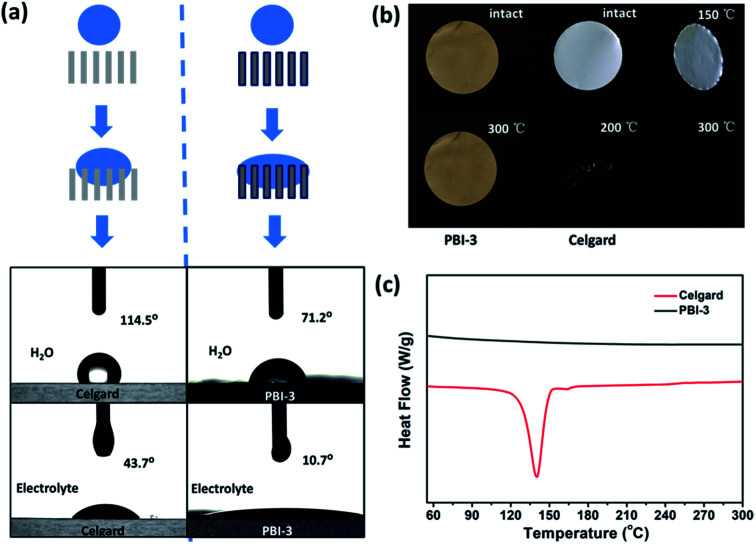
(a) Illustration of the wetting process and contact angle of Celgard membrane and PBI-3 membrane; (b) photographs of PBI-3 membrane and Celgard membrane after thermal treatments; (c) comparison of the DSC spectra of the PBI-3 membrane and the Celgard membrane.

The as-synthesized PBI-3 membrane shows the porosity and electrolyte uptake of about 76.81% and 286% (shown in Table S1†), which still much higher than that of Celgard membrane (43%, 92%). Fig. S4† shows the mechanical strength of PBI-3 membrane. Despite the PBI-3 membrane possesses the high porosity, it still retains good mechanical property with a tensile strength of 21.74 MPa, which is acceptable or superior to the membranes with a similar porosity.^45,54^ The excellent hydrophilic performance of PBI-3 membrane can increase the speed of the wetting process, as shown in the illustration of Fig. 4a. The contact angle measurement is performed to further evaluate the wettability of separator. The contact angles of as-prepared PBI-3 membrane are 71.2° and 10.7° towards water and electrolyte, which are markedly smaller than that (114.5° and 43.7° towards water and electrolyte) of Celgard separator. The satisfying result indicates that the PBI-3 membrane shows better wettability than Celgard membrane. The good wettability of the as-prepared PBI-3 membrane can attribute to the strongly polar nitrogen atoms of imidazole ring of the PBI main chain, further increasing the electrochemical performance of LIB. The inherent hydrophilic performance of PBI membrane is remarkable, as compared to the hydrophilic treatment process of introducing the inorganic nanoparticles for modifying separator in industrial production.

**Fig. 5:**
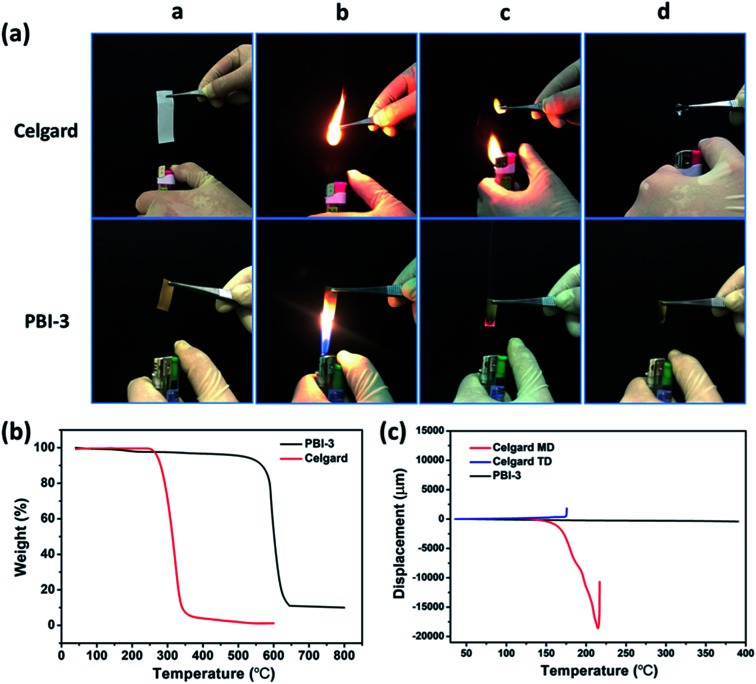
(a) The flame retarding behavior of Celgard membrane and PBI-3 membrane; (b) TGA curves of the as-prepared PBI-3 membrane and Celgard membrane in air atmosphere; (c) TMA curves of the PBI-3 membrane and the Celgard membrane.

The thermal dimensional stability of separator is crucial for LIB safety, particularly at elevated temperatures for high-performance batteries. Considering the safety issues of cell, therefore, the thermal dimensional stability of PBI-3 membrane is investigated before and after heating, and Celgard membrane is also evaluated as a comparison. The Celgard separator suffers severe thermal shrinkage at 150 °C and even totally degrades at 300 °C, which attributes to the low melting point of polyolefin (shown in Fig. 4b). Whereas, the PBI-3 membrane doesn't display any thermal shrinkage at an elevated temperature around 300 °C due to the existence of unique aromatic groups of PBI, revealing the superb thermal dimensional stability of PBI-3 membrane. Considering the ignitability of polyolefin separator under high-temperature, we have investigated the PBI porous membrane at elevated temperature. To further verify the fire resistance, the ignition tests is conducted on the PBI-3 membrane and Celgard membrane (Fig. 5a). When approaching fire, the Celgard membrane shrinks immediately and catches on fire in a short time. As expected, the PBI-3 membrane shows excellent self-extinguishing ability, proving the tremendous application potential as advanced LIBs separator. The DSC analysis of the PBI-3 and Celgard membranes are revealed in Fig. 4c, no endothermic peak is observed to match the polymer melting for the PBI-3 membrane in the whole scanning range, while the endothermic peak at 140 °C is found in the DSC curve of Celgard membrane. This result clearly indicates the satisfying thermal-stability of PBI-3 separator. As shown in Fig. 5b, the PBI-3 separator exhibits two obvious weight losses. The initial weight loss has occurred from 25 °C to 200 °C because of the evaporation of the absorbed water and the residual solvents within the membranes. Whereas the second weight loss is displayed at about 545 °C, attributing to the decomposition of the PBI main chain. The Celgard membrane, however, reveals sharp fall from 250 °C due to the polyolefin skeleton degradation. Hence, the outstanding thermal-stability demonstrates the PBI-3 membrane can be used in high temperature for LIBs separator. We have carried out the TMA test to evaluate the thermal dimensional stability. As shown in Fig. 5c, an obvious shrinkage is observed at MD direction about the Celgard membrane, and it presents no shrinkage in size until nearly 180 °C on the TD direction. In contrast, PBI-3 membrane doesn't appear any shrinkage even at an elevated temperature around 300 °C. Therefore, the PBI-3 membrane possessing outstanding thermal-stability and flame retardancy can be conducive to improve the safety of the LIBs.

**Fig. 6:**
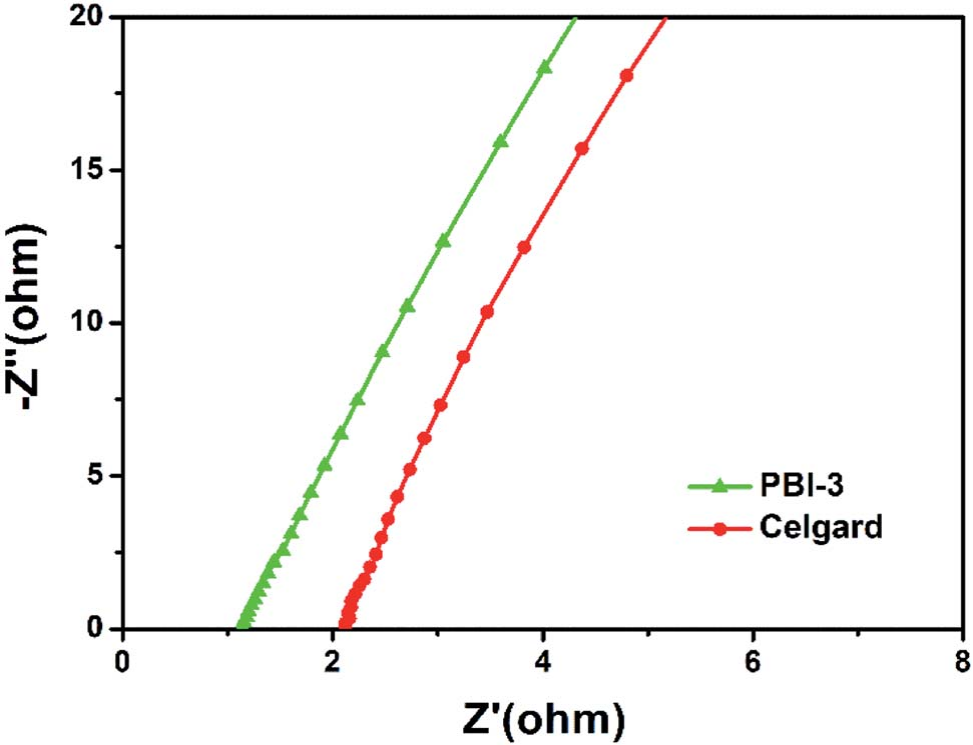
Nyquist plots of the cells SS/separator/SS using PBI-3 and celgard membranes, respectively.

The ionic conductivity of separator is investigated by electrochemical impedance spectroscopy. Fig. 6 presents the Nyquist plots of the electrochemical impedance for the PBI-3 membrane and Celgard membrane in liquid electrolyte. Also, it is observed that the imaginary part of impedances is proportion to their real part at the low frequency range. Besides, the resistance of separator in liquid electrolyte can be evaluated by the junction of linear relation between the imaginary and real parts of the impedance with the real part axis. The ionic conductivity of the PBI-3 membrane and Celgard membrane is calculated to be 1.16 mS cm^−1^ and 0.59 mS cm^−1^ using the eqn (3), implying that the PBI-3 membrane owns better ionic conductivity than that of Celgard membrane. The result can attribute to the existence of good electrolyte uptake and hydrophilicity of the PBI. Meanwhile, the polar groups of N–H bonds can offer functional sites for the efficient adhesion and homogeneous distribution of Li ions, which can facilitate the lithium-ions transport of electrolyte-immersed separator.

**Fig. 7:**
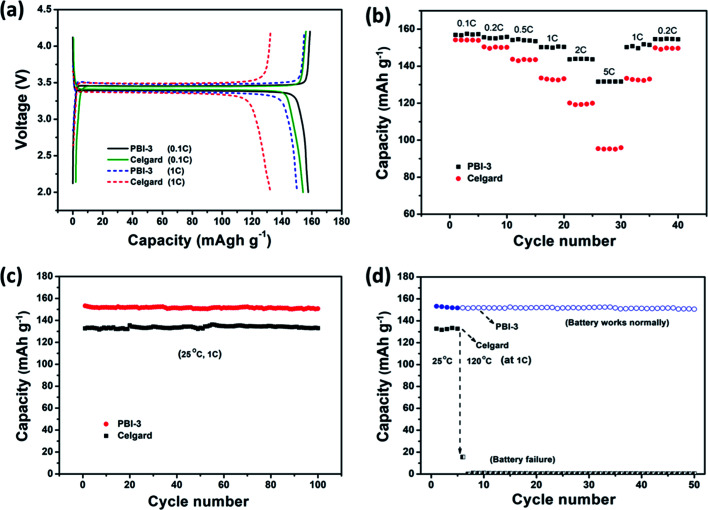
(a) The charge–discharge curves of batteries with PBI-3 and Celgard-2400 membranes at 0.1C rate and 1C rate; (b) rate capability of PBI-3 and Celgard membranes; (c) cycle performance of cells at 1C at 25 °C; (d) cycle performance of cells at 1C at 25 °C and 120 °C.

The charge–discharge curves of cells using the PBI-3 and Celgard separators were analysed at C-rates of 0.1C and 1C, as shown in Fig. 7a. The batteries assembled with PBI-3 membrane shows the typical and stable charge and discharge potential plateaus. As anticipated, the PBI-3 membrane shows higher capacity at C-rates of 0.1 and 1C as compared to Celgard membrane. The discharge capacity of the cells using the PBI-3 and Celgard separators are about 157.6 and 154.2 mA h g^−1^ at 0.1C and 150.3 and 132.5 mA h g^−1^ at 1C, respectively. The cells using the PBI-3 separators display the higher charge–discharge property at 1C as compared to the Celgard separator, which attributes to the superiority of high electrolyte uptake and outstanding wettability of PBI spongelike porous membrane. Meanwhile, the rate capability of the cells using PBI-3 separator is further investigated by comparing the Celgard separator at various charge–discharge densities. As shown in Fig. 7b, the discharge capacity of the cells using the PBI-3 and Celgard separators are about 157.1 mA h g^−1^ and 154.2 mA h g^−1^ at 0.1C. With the increase of the charge discharge density, the PBI-3 separator shows higher capacity retention than Celgard separator. The cell using the PBI-3 separator shows the discharge capacities of 155.6 mA h g^−1^ at 0.2C, 154.1 mA h g^−1^ at 0.5C, 150.4 mA h g^−1^ at 1C, 143.9 mA h g^−1^ at 2C, and 131.7 mA h g^−1^ at 5C, respectively. Additionally, the discharge capacity of the cell using the Celgard separator are about 150.3 mA h g^−1^ at 0.2C, 143.5 mA h g^−1^ at 0.5C, 133.6 mA h g^−1^ at 1C, 119.5 mA h g^−1^ at 2C, and 95.4 mA h g^−1^ at 5C, respectively. Compared with the Celgard membrane, the cells using the PBI-3 membranes present stable and perfect battery performance than that of Celgard separator in different charge–discharge densities, especially at higher charge–discharge density (5C), which attributes to the high porosity and high electrolyte wettability of PBI-3 membrane. Fig. 7c shows the cycle performance at 1C rate for 100 cycles. The discharge capacity of the cells assembled with PBI-3 and Celgard membranes displays stable cycling performance after 100 cycles, which has a little disturbance because of the minor changes of the environment. Besides, the cycling performance is also investigated at high temperature, as presented in Fig. 7d. The battery with the PBI-3 membrane can work normally at a temperature of 120 °C and present good cycling stability, attributing to the superb thermal-stability of PBI. Nevertheless, the battery with a Celgard membrane immediately fails when the temperature was raised to 120 °C. The result clearly demonstrates that the PBI-3 membrane can be used as the new generation high-safety and high-performance LIBs.

## Conclusion

In this work, we have successfully designed a new, flexible, and controllable approach for the fabrication of the multifunctional PBI porous membrane by simply extracting imidazole from PBI blend membranes with deionized water. The PBI porous membrane displays exciting comprehensive properties with excellent wettability, superb fire-resistance, outstanding thermal dimensional stability, and superior electrochemical performance. Additionally, the existence of plentiful polar groups of PBI can regulate the Li^+^ flux and improve the ionic conductivity. Notably, the cell with the as-synthesized PBI porous membrane presents satisfying rate capability with 84% capacity retention from 0.1C (157.1 mA h g^−1^) up to 5C (131.7 mA h g^−1^) as compared to the Celgard separator, and it can keep stable cycling performance and maintain normal work at a temperature of 120 °C. More importantly, the as-prepared PBI porous membranes can improve the thermal safety issues owing to the outstanding flame resistance and thermal dimensional stability. Even though more methods have been established for fabricating the separators, we anticipate that the high-efficiency and eco-friendly strategy can point to an interesting and new direction for the next generation separator, leading to a breakthrough for high-safety and high-performance LIBs.

## Conflicts of interest

There are no conflicts to declare.

## Supplementary Material

RA-009-C9RA08006F-s001
