# Supplementary material for: The design of a multifunctional separator regulating the lithium ion flux for advanced lithium-ion batteries
Source: RSC Adv. 2019 Dec 4;9(68):40084–91. doi: 10.1039/c9ra08006f (PMC9076257; doi:10.1039/c9ra08006f)
Supplement: RA-009-C9RA08006F-s001 [file RA-009-C9RA08006F-s001.pdf]

Electronic Supplementary Material (ESI) for RSC Advances.
This journal is © The Royal Society of Chemistry 2019

Supporting information for:

The design of multifunctional separator regulating the lithium ion flux for advanced lithium-ion batteries

Guohua Sun[a], Jiacong Guo[a], Hongqing Niu[a], Nanjun Chen[c], Mengying Zhang[a], Guofeng Tian[a], Shengli Qi[a,b]*, Dezhen Wu[a]**

[a] State Key Laboratory of Chemical Resource Engineering, Beijing University of Chemical Technology, Beijing 100029, China.

[b] Changzhou Institute of Advanced Materials, Beijing University of Chemical Technology, Changzhou 213164, Jiangsu, China.

[c] Department of Energy Engineering, College of Engineering, Hanyang University, Seoul 04763, Republic of Korea.

* Corresponding author: Prof. Shengli Qi. Tel.: +86 010 6442 2381;
E-mail addresses: qisl@mail.buct.edu.cn (Shengli Qi)

** Corresponding author: Prof. Dezhen Wu. Tel.: +86 010 6442 1693;
E-mail addresses: wdz@mail.buct.edu.cn (Dezhen Wu)

Table of content

Fig. S1 The process for synthesis of PBI polymer.

Fig. S2 The ^{1}H NMR of the synthesized PBI polymer.

Fig. S3 The FTIR spectrum of the synthesized PBI polymer.

Table. S1 Physical Properties of PBI-3 and Celgard Separators

Fig. S4 The stress-strain curve of PBI-3 membrane.

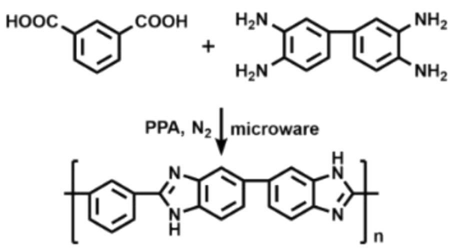

Fig. S1 The process for synthesis of PBI polymer.

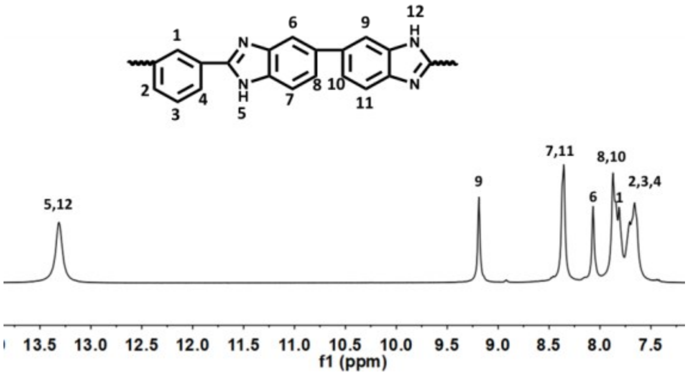

Fig. S2 The ^1H NMR of the synthesized PBI polymer.

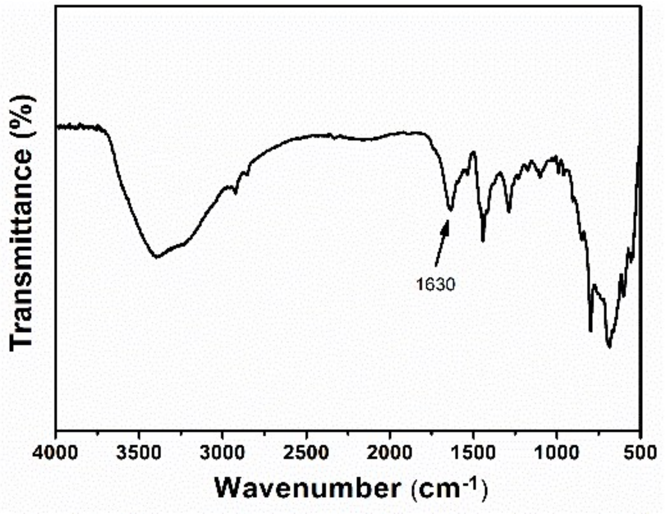

Fig. S3 The FTIR spectrum of the synthesized PBI polymer.

Table. S1 Physical Properties of PBI-3 and Celgard Separators

Sample	Thickness (μm)	Porosity (%)	electrolyte contact angle (deg)	Electrolyte uptake (%)
PBI-3	45	76.81	43.7	286
Celgard	25	43	10.7	92

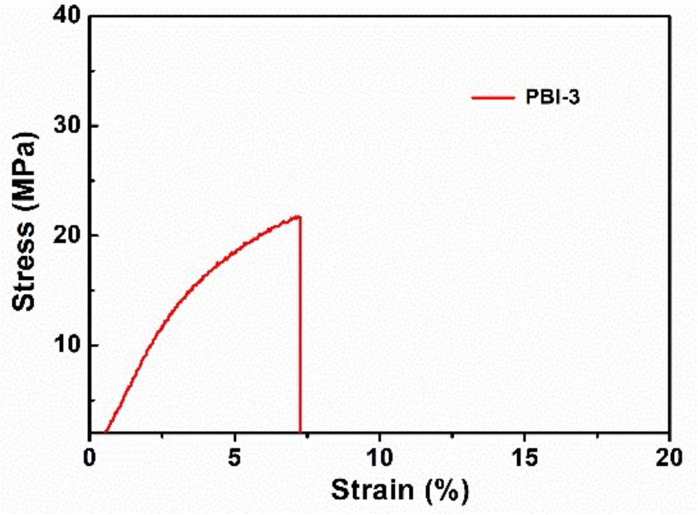

Fig. S4 The stress-strain curve of PBI-3 membrane.
